# AI for Analyzing Mental Health Disorders Among Social Media Users: Quarter-Century Narrative Review of Progress and Challenges

**DOI:** 10.2196/59225

**Published:** 2024-11-15

**Authors:** David Owen, Amy J Lynham, Sophie E Smart, Antonio F Pardiñas, Jose Camacho Collados

**Affiliations:** 1 School of Computer Science and Informatics Cardiff University Cardiff United Kingdom; 2 Centre for Neuropsychiatric Genetics and Genomics, Division of Psychological Medicine and Clinical Neurosciences School of Medicine Cardiff University Cardiff United Kingdom

**Keywords:** mental health, depression, anxiety, schizophrenia, social media, natural language processing, narrative review

## Abstract

**Background:**

Mental health disorders are currently the main contributor to poor quality of life and years lived with disability. Symptoms common to many mental health disorders lead to impairments or changes in the use of language, which are observable in the routine use of social media. Detection of these linguistic cues has been explored throughout the last quarter century, but interest and methodological development have burgeoned following the COVID-19 pandemic. The next decade may see the development of reliable methods for predicting mental health status using social media data. This might have implications for clinical practice and public health policy, particularly in the context of early intervention in mental health care.

**Objective:**

This study aims to examine the state of the art in methods for predicting mental health statuses of social media users. Our focus is the development of artificial intelligence–driven methods, particularly natural language processing, for analyzing large volumes of written text. This study details constraints affecting research in this area. These include the dearth of high-quality public datasets for methodological benchmarking and the need to adopt ethical and privacy frameworks acknowledging the stigma experienced by those with a mental illness.

**Methods:**

A Google Scholar search yielded peer-reviewed articles dated between 1999 and 2024. We manually grouped the articles by 4 primary areas of interest: datasets on social media and mental health, methods for predicting mental health status, longitudinal analyses of mental health, and ethical aspects of the data and analysis of mental health. Selected articles from these groups formed our narrative review.

**Results:**

Larger datasets with precise dates of participants’ diagnoses are needed to support the development of methods for predicting mental health status, particularly in severe disorders such as schizophrenia. Inviting users to donate their social media data for research purposes could help overcome widespread ethical and privacy concerns. In any event, multimodal methods for predicting mental health status appear likely to provide advancements that may not be achievable using natural language processing alone.

**Conclusions:**

Multimodal methods for predicting mental health status from voice, image, and video-based social media data need to be further developed before they may be considered for adoption in health care, medical support, or as consumer-facing products. Such methods are likely to garner greater public confidence in their efficacy than those that rely on text alone. To achieve this, more high-quality social media datasets need to be made available and privacy concerns regarding the use of these data must be formally addressed. A social media platform feature that invites users to share their data upon publication is a possible solution. Finally, a review of literature studying the effects of social media use on a user’s depression and anxiety is merited.

## Introduction

### Background

The Global Burden of Disease study (1990-2019) reports that anxiety disorders, major depressive disorder, and schizophrenia are the main drivers of years lived with disability and disability-adjusted life years across all age groups worldwide [1]. These mental health conditions are a sizable burden on the global population and public health systems. To help alleviate these problems, early intervention is essential [2].

The experiences of those with mental health disorders are often recounted on social media [3]. More broadly, users of Facebook and Reddit express favorable and adverse life events through the medium of text [4,5], and pictorial expressions of sensitive topics such as illness or hardship are becoming increasingly common through image-focused platforms such as Instagram [6]. As a result, methods that harness social media data for prediction of the mental health status of users have burgeoned [7-9]. Research has also spiked following the COVID-19 pandemic [10] and has become a truly interdisciplinary pursuit involving not only computer scientists but also psychologists, psychiatrists, and neuroscientists [11]. The broad idea behind this field is that models underpinned by artificial intelligence (AI) can “predict” a person’s “mental health status” (refer to the study by Chancellor et al [12] for a discussion on the meaning of these terms in this literature). A branch of AI that is most appropriate for these methods is natural language processing (NLP), which uses computational techniques to learn, understand, and produce human language content [13]. Text-based dialogue systems, for example, have become a mainstay of NLP research. Their use in assisting people with neurocognitive disorders or mental health conditions is a popular application area. An early system, ELIZA [14], dates back to 1966. It purported to perform the role of a psychotherapist in conversation with a patient and has influenced the design of modern conversational agents such as ChatGPT [15]. In 2024, the potential for adults with dementia to adopt ChatGPT as a memory aid has been explored; it may be able to provide reminders of names, dates, and events, thus easing anxiousness [16]. The mining of text data to help assess a person’s mental state has also followed from pre–21st century work. The *Whissell Dictionary of Affect in Language* [17], compiled in 1989 and now available on the web [18], can be used to estimate the mood conveyed in a body of text. This has given rise to modern methods for predicting the mental health status of social media users. Indeed, the huge volume of human language content available on the web, for example in Facebook and Reddit postings, fits very well the technical constraints of NLP techniques and can be straightforwardly processed into model inputs.

Some of the earliest attempts at predicting the mental health statuses of members of web-based communities were done without AI, through manual review of postings and classic statistical analyses. For example, in November 1999, psychiatrists monitored the general psychiatry subforum of the Norwegian web-based forum Doktoronline [19,20]. They observed that users who wrote negatively about their mental health by expressing sadness or resignation typically received positive and constructive responses from other users. Subsequently, these users often sought social support in their local communities. This corroborated previous findings showing that web-based community participation can have positive, real-life consequences for individuals [21,22], a motivation for later attempts at developing automatic health care intervention methods. Haker et al [23] examined the writings of web-based forum users who self-disclosed diagnoses of schizophrenia. They too noted that users with schizophrenia benefited by receiving advice from other users about medications and approaching health care professionals, as well as by receiving empathy and support.

The advent of social media platforms such as Facebook provided further locations for discussion about mental health disorders. Moreno et al [24] recognized that instances of major depressive disorder (depression hereafter) can be challenging to identify, particularly in older adolescents. So, between 2009 and 2010 they sought Facebook profiles of freshman students whose status updates referenced depression symptoms. Such students were then contacted and those who were willing were clinically screened to determine a diagnosis of depression. Students displaying depression symptoms in their status updates were more than twice as likely to be at risk for depression. Furthermore, the status updates referencing depression symptoms were often found to be a means of gathering support or attention, yet the students showed reluctance in seeking help in person. Thus, it was recognized that Facebook depression disclosures could be harnessed to identify those who might have unmet needs for mental health care. This provided an explicit motivation for improving the methods for predicting this disorder early in its course.

### This Study

Due to the large volume of literature that exists in this area, which swelled during the COVID-19 pandemic, a review is timely. In this study, we focused on methods that concern the detection of language features presented in the texts of user social media postings. The main aim of our review was to ascertain state-of-the-art methodologies for detecting linguistic features that can be attributed to mental illnesses. This includes cataloging datasets containing “ground truth” (gold standard) labels of mental health status [12], which are available to help fine-tune these methodologies. Ground truths may be obtained from electronic health records (EHRs), clinical questionnaires, or self-disclosure statements of a mental health diagnosis (eg, “I was diagnosed with depression”). We then examined how these methodologies integrate the temporal stochasticity of mental states as reflected by longitudinal studies. We also identified common technical and ethical constraints met in the development of the reviewed studies. Finally, we will form recommendations for the future direction of AI-based research on mental health.

## Methods

### Overview

We used Google Scholar to seek peer-reviewed articles published between January 1999 and February 2024. This literature search engine was selected because it is considered the most comprehensive search engine in academia [25-27]. It offers particularly extensive coverage of computer science and informatics, which is the primary discipline of literature that forms this review, outperforming databases like Scopus [28]. Our search aimed to retrieve literature covering the 3 main mental health burdens reported by the Global Burden of Disease study [1]: depression, anxiety, and schizophrenia, which are all common mental disorders. The articles then underwent a manual selection exercise to assign each of them to 1 of the 4 different subject areas that cover important and distinct aspects around mental health research in social media: datasets on social media and mental health, methods for predicting mental health status, longitudinal analyses of mental health, and ethical aspects of the data and analysis of mental health. These subject areas, described in more detail in Textbox 1, underpin the aims of this review described in the Introduction section.

**The subject areas covered in this narrative review.**

**Datasets on social media and mental health**

To develop methods for predicting mental health status or conducting longitudinal analyses, carefully constructed social media datasets are required. We identify publicly available datasets that support this work and the challenges met in constructing them.

**Methods for predicting mental health status**

Approaches may consider how to detect mental health disorders in social media users and measure attributes of those disorders, such as their severities. We examine the progress in this area against a backdrop of evolving natural language processing technologies.

**Longitudinal analyses of mental health**

One’s mental health state is fluid. We review attempts to gauge mental health state changes at both an individual level and population level. The former may assist in directing personalized health care to people at risk, while the latter may help inform public health policy.

**Ethical aspects of the data and analysis of mental health**

Research activities in the domain of predicting mental health status inevitably involve the acquisition and processing of personal data. We study the concerns reported among the general population and how they may be ameliorated.

### Literature Search and Selection Strategy

A detailed exposition of the literature search and selection strategy, which is informed by Ferrari [29], now follows.

The search string in Textbox 2 was deployed to search article titles. 4479 articles were returned.

**Figure 1:**
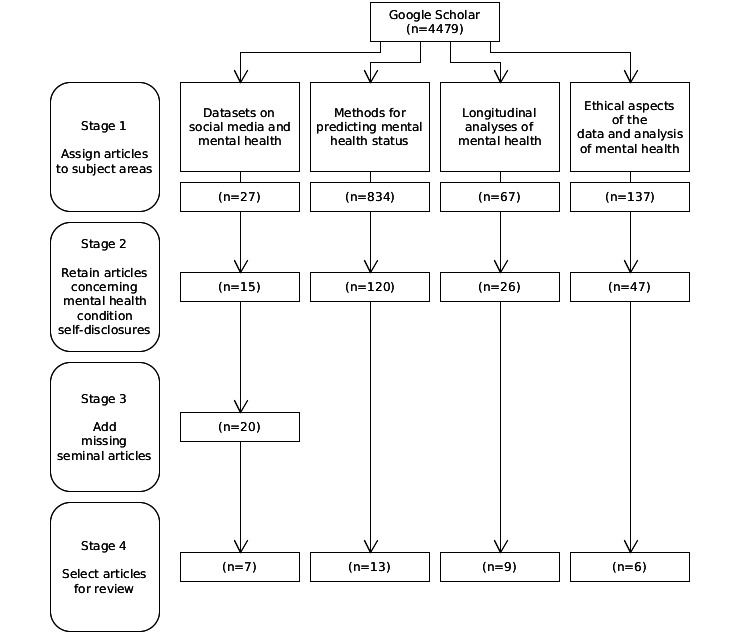
The 4-stage sifting process for selecting articles for inclusion in the narrative review.

The articles then underwent a 4-stage manual sifting exercise as shown in Figure 1.

**Search string used in Google Scholar.**

(intitle:“mental health”

OR intitle:“mental illness”

OR intitle:“mental disorder”

OR intitle:“psychiatric disorder”

OR intitle:“depression”

OR intitle:“anxiety”

OR intitle:“schizophrenia”)

AND

(intitle:“social media”

OR intitle:“forum”

OR intitle:“forums”

OR intitle:“facebook”

OR intitle:“twitter”

OR intitle:“reddit”)

In the first stage, the title and abstract of each article was inspected so that it could be assigned to 1 of the 4 different subject areas shown in Textbox 1. Articles that did not relate to any of the 4 areas of interest were discarded. Duplicate results, preprints, presentation slides, posters, and non–English language articles were also discarded. A total of 1065 articles remained after this stage.

In the second stage, bodies of candidate articles for the 4 subject areas underwent inspection. Only articles where the participants of the studies self-disclosed a diagnosis of depression, anxiety, or schizophrenia were retained. The purpose was to ensure that only studies that used this ground truth were considered.

A third stage was performed that affected only the datasets on social media and mental health subject area. Due to our inherent knowledge of the subject area, we recognized that following the second stage, 5 seminal papers were absent. These articles were not retrieved in the Google Scholar search (Textbox 2). Their omission appeared due to their titles containing only implicit references to social media platforms. For example, the article “RSDD-Time: Temporal Annotation of Self-Reported Mental Health Diagnoses” does not explicitly mention that it concerns social media data. Following completion of the third stage, we arrived at 20 articles in this subject area.

A fourth and final stage involved selecting the articles for review in each subject area. The selection process identified the most pertinent articles over a broad timespan that concerned depression, anxiety, and schizophrenia.

A comprehensive listing of the articles considered at each stage of the exercise is provided in Multimedia Appendix 1.

## Results

### Overview

Following the 4-stage manual sifting exercise, 35 articles across the 4 subject areas were finally selected for review. The content of these articles covered research activity undertaken between 1999 and 2024 and influential events such as the COVID-19 pandemic. Table 1 describes the articles that were finally included for review. The format of this table is drawn from Szeto et al [30]. A narrative review of these articles is presented in the following 4 sections, which cover each of our 4 subject areas.

**Table 1:** Articles across the 4 subject areas that were selected for review.

Subject area and article title		Year published	Study population	Summary
**Subject area: datasets on social media and mental health**				
	Predicting Depression via Social Media [31]	2013	Posts by 476 Twitter users who self-reported a diagnosis of depression between September 2011 and June 2012	Development of methods for dataset construction via crowdsourcing and quantifying users’ depressive language use during the year before their diagnosis
	Quantifying Mental Health Signals in Twitter [32]	2014	Posts published between 2008 and 2013 by 6696 Twitter users with a self-stated diagnosis of a mental health disorder: 394 with bipolar disorder, 441 with depression, 244 with PTSD^a^, 159 with seasonal affective disorder, and 5728 controls	Development and evaluation of a method for swift and inexpensive capture of data about a range of mental illnesses
	Depression and Self-Harm Risk Assessment in Online Forums [33]	2017	Posts published between January 2006 and October 2016 by 9210 Reddit users with a self-stated diagnosis of depression and 107,274 controls	Development and evaluation of a method for recognizing users with depression from their language use alone
	RSDD-Time: Temporal Annotation of Self-Reported Mental Health Diagnoses [34]	2018	Self-reported depression diagnosis posts by 598 Reddit users published between June 2009 and October 2016	Development of methods for rule-based time extraction of depression diagnosis dates and mental health condition state classification
	SMHD: A Large-Scale Resource for Exploring Online Language Usage for Multiple Mental Health Conditions [35]	2018	Posts published between January 2006 and December 2017 by 385,476 Reddit users with a self-stated diagnosis of a mental health disorder: 10,098 users with ADHD^b^, 8783 users with anxiety, 2911 users with autism, 6434 users with bipolar disorder, 14,139 users with depression, 598 users with eating disorders, 2336 with OCD^c^, 2894 with PTSD, 1331 with schizophrenia, and 335,952 controls	Development of methods for recognizing self-reported mental health condition diagnoses and obtaining high-quality labeled data automatically, rather than manually
	Mental Health Surveillance over Social Media with Digital Cohorts [36]	2019	Randomly selected posts belonging to 48,000 US Twitter users	Development of methods for automatically inferring characteristics, including gender, ethnicity, and location of randomly collected Twitter users
	Overview of eRisk at CLEF 2021: Early Risk Prediction on the Internet (Extended Overview) [37]	2021	Posts published between November 2009 and October 2020 by 80 Reddit users who completed a BDI-II^d^ questionnaire	Development of methods for determining the severity of depression in Reddit users
**Subject area: methods for predicting mental health status**				
	Social Media as a Measurement Tool of Depression in Populations [3]	2013	Posts by 117 Twitter users who indicated that they have clinical depression with onset between September 2011 and June 2012 and 157 controls	Development of methods for determining a social media depression index that may serve to gauge levels of depression in populations
	Beyond LDA: Exploring Supervised Topic Modeling for Depression-Related Language in Twitter [38]	2015	Posts by approximately 2000 Twitter users, of whom approximately 600 self-identified as having been clinically diagnosed with depression	Investigation into the use of topic models to analyze linguistic signals for detecting depression
	Quantifying the Language of Schizophrenia in Social Media [39]	2015	Posts published between 2008 and 2015 by 174 Twitter users who self-reported a diagnosis of schizophrenia	Development of methods for analyzing how the language of schizophrenia can aid in identifying and getting help to people with schizophrenia
	Recognizing Depression from Twitter Activity [40]	2015	CES-D^e^ questionnaire responses and posts by 209 Twitter users	Development of methods for extracting and using features from the activity histories of Twitter users to estimate the presence of depression
	A Collaborative Approach to Identifying Social Media Markers of Schizophrenia by Employing Machine Learning and Clinical Appraisals [41]	2017	Posts published between 2012 and 2016 by 146 Twitter users who self-disclosed a diagnosis of schizophrenia and 146 controls	Development of methods for combining linguistic features of Twitter content with clinical appraisals to form a diagnostic tool for identifying individuals with schizophrenia
	Detecting depression and mental illness on social media: an integrative review [42]	2017	43 peer-reviewed articles	Literature review of methods for predicting mental illness using social media
	Forecasting the onset and course of mental illness with Twitter data [43]	2017	Posts by 105 Twitter users who had a diagnosis of depression and 99 controls. Also, posts by 174 Twitter users who had a diagnosis of PTSD	Development of models to predict the emergence of depression in Twitter users
	A text classification framework for simple and effective early depression detection over social media streams [44]	2019	Posts by 135 Reddit users who have depression and 752 controls	Development of a text classification approach for early risk detection concerning social media users with depression, with an emphasis on explainable AI^f^
	Towards Preemptive Detection of Depression and Anxiety in Twitter [45]	2020	Posts by 548 Twitter users who self-disclosed having either depression or anxiety and 4102 controls	Development of an LM^g^-based approach for early detection of depression in Twitter users
	A Transformers Approach to Detect Depression in Social Media [46]	2021	Posts by 4000 Reddit users who self-disclosed having depression and 4000 controls	Development of transformer-based models for detecting depression in social media users
	Characterisation of Mental Health Conditions in Social Media Using Deep Learning Techniques [47]	2022	77 peer-reviewed articles	Literature review of research concerning DL^h^ techniques for identifying various mental health conditions from social media data
	Utilizing ChatGPT Generated Data to Retrieve Depression Symptoms from Social Media [48]	2023	Posts by 3107 Reddit users	Development of methods for generating synthetic social media data for subsequent use in transformer-based language model depression detection
	Prompt-based mental health screening from social media text [49]	2024	Posts by 1684 Twitter users who self-reported a diagnosis of depression and 11,788 controls	Development of methods that use LLM^i^ prompting as an aid to mental health screening in social media text
**Subject area: longitudinal analyses of mental health**				
	Feeling bad on Facebook: Depression disclosures by college students on a social networking site [24]	2011	Facebook profiles of 200 university students	Development of methods for determining associations between displayed depression symptoms on Facebook and other demographic or Facebook use characteristics
	Towards Assessing Changes in Degree of Depression through Facebook [50]	2014	Status updates and survey responses of 28,749 Facebook users collected between June 2009 and March 2011	Development of a regression model to predict users’ degrees of depression based on their Facebook status updates
	Small but Mighty: Affective Micropatterns for Quantifying Mental Health from Social Media Language [51]	2017	Posts by 3680 Twitter users with a self-stated diagnosis of a mental health condition: 2271 with generalized anxiety disorder, 687 with eating disorders, 247 prone to panic attacks, 318 with schizophrenia, and 157 who have attempted suicide	An investigation of textual patterns in Tweet sequences occurring over short time windows to ascertain their suitability in quantifying psychological phenomena
	Monitoring Online Discussions About Suicide Among Twitter Users With Schizophrenia: Exploratory Study [52]	2018	Posts by 203 Twitter users who self-identified as having schizophrenia and 173 controls	An exploration of the feasibility of monitoring web-based discussions about suicide among Twitter users who self-identify as having schizophrenia
	Predicting Depression From Language-Based Emotion Dynamics: Longitudinal Analysis of Facebook and Twitter Status Updates [53]	2018	Status updates and depression severity ratings of 29 Facebook users and 49 Twitter users	A study of the associations between depression severity and emotion word expression on Facebook and Twitter status updates
	What about Mood Swings: Identifying Depression on Twitter with Temporal Measures of Emotions [54]	2018	Posts by 585 Twitter users who self-reported a diagnosis of depression and 6596 controls	Development of a method for identifying users with or at risk of depression by incorporating measures of 8 emotions as features from Twitter posts over time, including a temporal analysis of these features
	Monitoring Depression Trends on Twitter During the COVID-19 Pandemic: Observational Study [55]	2021	Posts of 2575 Twitter users who self-disclosed a diagnosis of depression and 2575 controls	Development of transformer-based DL language models to identify users with depression from their everyday language and to monitor the fluctuation of their depression levels
	Using language in social media posts to study the network dynamics of depression longitudinally [56]	2022	Posts by 946 Twitter users who self-reported the dates of any depressive episodes in the past 12 months and the severity of their current depressive symptoms	An investigation into the association between depression severity and text features in Twitter posts
	Enabling Early Health Care Intervention by Detecting Depression in Users of Web-Based Forums using Language Models: Longitudinal Analysis and Evaluation [57]	2023	Posts by 56 Reddit users who self-reported a diagnosis of depression and 168 controls	An investigation to determine the time points in the posting history of a person with depression, that are most indicative of their depression
**Subject area: ethical aspects of the data and analysis of mental health**				
	Effectiveness of Social Media Interventions for People With Schizophrenia: A Systematic Review and Meta-Analysis [58]	2016	2 peer-reviewed publications	Literature review of the effectiveness of social media interventions for supporting people with schizophrenia
	Ethical issues in using Twitter for population-level depression monitoring: a qualitative study [59]	2016	16 Twitter users with a self-reported diagnosis of depression who participated in a series of focus groups and 10 controls	Cross-sectional survey study of public attitudes toward using Twitter data for mental health monitoring
	Social media, big data, and mental health: current advances and ethical implications [60]	2016	62 peer-reviewed articles	Literature review of work that uses social media “big data,” NLP^j^, and ML^k^ for mental health surveillance and the ethical considerations therein
	Who is the “Human” in Human-Centered Machine Learning: The Case of Predicting Mental Health from Social Media [61]	2019	55 peer-reviewed articles	Literature review of how scientific articles represent human research participants in human-centered ML
	Ethics and Privacy in Social Media Research for Mental Health [62]	2020	35 peer-reviewed articles	Literature review of research that uses social media data in the context of mental health, with reference to the challenges in relation to consent, privacy, and use of such data
	Understanding the Role of Social Media–Based Mental Health Support Among College Students: Survey and Semistructured Interviews [63]	2021	101 US university students aged 18 to 24	Web-based survey followed by semistructured interviews to investigate into whether and how social media platforms help meet university students’ mental health needs in terms of the social support that they offer

^a^PTSD: posttraumatic stress disorder.

^b^ADHD: attention-deficit/hyperactivity disorder.

^c^OCD: obsessive-compulsive disorder.

^d^BDI-II: Beck Depression Inventory-II.

^e^CES-D: Center for Epidemiologic Studies Depression Scale.

^f^AI: artificial intelligence.

^g^LM: language model.

^h^DL: deep learning.

^i^LLM: large language model.

^j^NLP: natural language processing.

^k^ML: machine learning.

### Datasets on Social Media and Mental Health

To develop methods for predicting mental health status, access to high-quality datasets is essential. De Choudhury et al [31] observed in 2013 that previous research had relied heavily on small, homogeneous samples of individuals who gave retrospective self-reports about their mental health, often via surveys. The authors also recognized that a person’s posting activity on social media could provide time-stamped insights into their psychological state. To this end, they used crowdsourcing to compile a dataset of tweets belonging to 476 Twitter users who self-reported a diagnosis of depression. The data were subsequently used to analyze linguistic and behavioral patterns, such as symptom mentions and diurnal activity, respectively. While the data were deemed high quality by Coppersmith et al [32], they pointed to the limited size and scope of these data in terms of self-reported diagnoses, which needed to be obtained by manual completion of a questionnaire, namely the Center for Epidemiologic Studies Depression Scale screening test. Therefore, they proposed an automated method for labeled dataset construction, which sought self-reports of mental illness diagnoses on Twitter such as “I was diagnosed with depression.” Their yield of >5000 different users conveying such statements between 2008 and 2013 indicated that a low-cost and low-resource method for data collection was possible. However, the authors acknowledged some limitations. First, only Twitter users were captured, a sample not likely representative of the general population but in this sense, similar to other social media datasets. Second, it was not possible to verify that the self-stated diagnoses were genuine or captured the same psychopathology as clinical diagnoses. For example, population biobank data have shown self-reported depression to be less heritable (ie, less of its variance in the population can be attributed to genetic factors) than diagnostically ascertained depression [64]. Nevertheless, this approach has ostensibly provided the foundation for several publicly available and widely used mental health datasets.

Yates et al [33] developed the Reddit Self-reported Depression Diagnosis (RSDD) dataset, which contains the posting histories of 9210 users with a diagnosis of depression revealed by self-report statements, like the ones described earlier. Further populated with 107,274 users without depression for control purposes, RSDD has become an often-used resource in the development of methods for predicting depression [65-70]. It has also propagated the development of 2 sister datasets, RSDD-Time [34] and Self-reported Mental Health Diagnoses (SMHD) [35]. The former was conceived by MacAvaney et al [34] after recognizing that research had largely not examined the temporality of mental health diagnoses. They randomly selected 598 posts from the RSDD dataset that contained the self-reported diagnosis statement of a user with depression and manually annotated them to denote when the diagnosis occurred. Owen et al [57] successfully exploited RSDD and RSDD-Time in a longitudinal study that evidenced a relationship between selected time spans before diagnosis and the sentiment a user exhibits in their postings. However, because many of the annotations in RSDD-Time denote that the diagnosis dates of many of the users cannot be estimated with a reasonable degree of accuracy (eg, the user merely stated that their depression diagnosis occurred “in the past”), the findings were predicated on the posting histories of only 56 users with depression. This highlights a need for much larger datasets where the dates of depression diagnoses are denoted with a high degree of accuracy.

SMHD, meanwhile, was born out of a desire for datasets covering a broad range of mental health disorders. It provided a platform for the development of methods concerning not only depression [71,72] but also suicidal ideation [73], schizophrenia [74], and even multiclass experimental setups involving combinations of anxiety, eating disorders, attention-deficit/hyperactivity disorder, bipolar disorder, and posttraumatic stress disorder [7,75-77]. It was also intended that a wider range of higher positive predictive value patterns be used to collect a greater volume of users who were diagnosed. Such patterns detect diagnosis keywords relevant to each disorder, drawn from the *Diagnostic and Statistical Manual of Mental Disorders* [78]. As a result, SMHD contains 20,406 users who were diagnosed and 335,952 matched controls. Despite these strengths, RSDD and SMHD are limited in their scope because they do not include posts made in mental health subreddits. It is recognized that language used in dedicated mental health subreddits systematically differs from the rest of Reddit [79]. This, and the limitation that they used only simple text patterns such as “I was diagnosed with depression” to collect users with mental health disorders, must be consistently considered in research work as it may introduce a bias to any models developed [80].

Other biases also exist in social media data. For example, most social media platforms, including Facebook, Twitter, and Instagram, have more male users [81]. There is also evidence to suggest that people with higher levels of education and household income are more frequent social media users [82]. To address such biases and improve the representativeness of social media datasets, Amir et al [36] considered a cohort-based approach to dataset construction. That is, they developed a demographic inference pipeline, which sought Twitter users and identified their age, gender, ethnicity, and location to create a subsample that was representative of the wider population. They then leveraged an existing model [83] to ascertain the prevalence of depression and posttraumatic stress disorder across the 48,000 users collected. This is in contrast to identifying users based on self-reported diagnosis statement patterns, which as mentioned, is another potential source of bias. The authors proposed that such use of surveillance-based methods could aid the identification of population-level trends in disorder prevalence. However, they also acknowledged that proper evaluation of these patterns would require disentangling the ways in which social media datasets differ from representative samples of the underlying population. In any case, further development and adoption of surveillance-based methods are constrained by privacy and ethical considerations. For example, it would surely require the permission of social media users before their data could be automatically sought and analyzed en masse, particularly in relation to personally identifiable information (age, gender, ethnicity, and health status). We explore these matters in more depth in the Ethical Aspects of the Data and Analysis of Mental Health section.

Finally in this section, we mention the work of the eRisk Lab [37], which touched upon another important dimension in the support of methods for predicting mental health status. Their 2021 dataset, which comprised Reddit posting histories belonging to 80 users, was accompanied by ground truth data that can aid in the development of methods for gauging the severity of depression. Recorded against each user was a completed Beck Depression Inventory-II (BDI-II) questionnaire, which categorizes the severity of their depression (ranging from minimal to severe). While the dataset proved useful in designing methods for finding associations between language features in the users’ postings and their depression severities, the ground truth BDI-II questionnaires provided only the depression severities at the terminuses of the users’ posting histories. Because the state of one’s mental health is somewhat fluid [34], the dataset may contain users whose depression may have long passed. This is plausible given that one user in the dataset had a posting history spanning >10 years, although it should be noted that this is an anomaly, with the median posting history in the dataset being just >1 year. Furthermore, the dataset’s small size in terms of number of users is a major constraint [84,85]. This highlights the difficulty in obtaining copious ground truth data that are traditionally collected via confidential questionnaires.

Table 2 summarizes some important features of the datasets discussed in this section, including the platform, contents, compilation year, acquisition inquiries information, and article title.

**Table 2:** Datasets discussed in this review that may be obtained from their authors.

Dataset	Platform	Contents	Year compiled	Acquisition inquiries	Article
RSDD^a^	Reddit	116,484 users: 9210 with depression and 107,274 controls	2014	RSDD dataset [86]	Depression and Self-Harm Risk Assessment in Online Forums [33]
RSDD-Time	Reddit	598 users with depression	2018	ir@Georgetown—resources [87]	RSDD-Time: Temporal Annotation of Self-Reported Mental Health Diagnoses [34]
SMHD^b^	Reddit	385,476 users: 10,098 with ADHD^c^, 8783 with anxiety, 2911 with autism, 6434 with bipolar disorder, 14,139 with depression, 598 with eating disorders, 2336 with OCD^d^, 2894 with PTSD^e,^ 1331 with schizophrenia, and 335,952 controls	2018	ir@Georgetown—resources—SMHD [88]	SMHD: A Large-Scale Resource for Exploring Online Language Usage for Multiple Mental Health Conditions [35]
2015 Computational Linguistics and Clinical Psychology Shared Task	Twitter	1746 users: 477 with depression, 396 with PTSD, and 873 controls	2015	CLPsych 2015 shared task evaluation [89]	Mental Health Surveillance over Social Media with Digital Cohorts [36]
eRisk 2021 Text Research Collection	Reddit	80 users who completed a BDI-II^f^ questionnaire	2021	eRisk 2021 text research collection [90]	Overview of eRisk at CLEF 2021: Early Risk Prediction on the Internet (Extended Overview) [37]

^a^RSDD: Reddit Self-reported Depression Diagnosis.

^b^SMHD: self-reported mental health diagnoses.

^c^ADHD: attention-deficit/hyperactivity disorder.

^d^OCD: obsessive-compulsive disorder.

^e^PTSD: posttraumatic stress disorder.

^f^BDI-II: Beck Depression Inventory-II.

### Methods for Predicting Mental Health Status

#### Background

The methods covered in this review are supported by machine learning (ML). As there is a broad terminology concerning ML, we introduce the relevant terms in Table 3.

**Table 3:** Machine learning (ML) terms used in this review.

Term		Description
**Data representation**		
	LDA^a^ [91]	A technique that can examine a group of documents and produce a series of words, known as a topic, that characterizes those documents. For example, “anatomy, dissection, genomes” may form the topic of a collection of biomedical documents.
	LIWC^b^ [92]	A text analysis technique that can infer the emotion conveyed in text (eg, positive or negative).
	Ontology [93]	A graphical representation of knowledge that is both human-readable and machine-readable. For example, a biomedical ontology might show how different neurological signs and symptoms may be linked to relevant diseases.
	Data augmentation [94]	The methods used to increase the size of a dataset by adding slightly modified copies of existing items in the dataset.
**Algorithms**		
	Supervised learning [95]	A type of ML^c^ algorithm analogous to human learning from past experiences to gain new knowledge to improve our ability to perform real-world tasks.
	SVM^d^ [96]	A supervised ML algorithm that learns by assigning labels to objects and can be used, for example, to recognize fraudulent credit card activity.
	Random forest [97]	A supervised ML algorithm that combines the output of multiple decision trees to reach a single result.
	DL^e^ [98]	A type of ML algorithm (supervised or unsupervised) that can produce complex models from data without features (eg, LIWC) needing to be derived as input.
**Pretrained models**		
	LM^f^ [99]	An LM is a probability distribution over words or word sequences. LMs learn to predict text that might come before and after other text and thus are used in tasks such as predicting text when writing an email.
	BERT^g^ [100]	An LM that examines words within text by considering both left-to-right and right-to-left contexts.
	ALBERT^h^ [101]	A lightweight alternative to BERT that is suitable for use where less computing power is available.
	MentalBERT [102]	An LM designed specifically to aid NLP^i^ tasks in the mental health care research community.
	MentalRoBERTa [102]	An alternative to MentalBERT that can perform predictions in longer left-to-right and right-to-left contexts.
	LLM^j^ [103]	Large-scale LM designed for NLP tasks such as producing complex text.
	GPT [104]	A family of neural network (in that they mimic the workings of the human brain) models that support AI^k^-driven applications for creating content such as text, images, or sound.
	ChatGPT [105]	A chatting robot that can provide a detailed response to a question or instruction.
**Performance metrics**		
	Positive predictive value [98]	Of the instances in a dataset predicted by an ML algorithm to have a certain label, positive predictive value denotes how many of them indeed have that label. This is often referred to as precision in the ML literature.
	Sensitivity [106]	Of the instances in a dataset with a particular label, sensitivity denotes how many of them were predicted correctly by an ML algorithm. Sensitivity is also known as recall.
	*F*_1_-score [107]	The harmonic mean of positive predictive value and sensitivity.
	AUROC^l^ [108]	Denotes an ML algorithm’s performance in terms of distinguishing between labels.

^a^LDA: latent Dirichlet allocation.

^b^LIWC: Linguistic Inquiry and Word Count.

^c^ML: machine learning.

^d^SVM: support vector machine.

^e^DL: deep learning.

^f^LM: language model.

^g^BERT: Bidirectional Encoder Representations from Transformers.

^h^ALBERT: A Lite Bidirectional Encoder Representations from Transformers.

^i^NLP: natural language processing.

^j^LLM: large language model.

^k^AI: artificial intelligence.

^l^AUROC: area under the receiver operating characteristic.

#### Traditional ML Approaches

In 2013, methods for predicting mental health status from social media data began to emerge [12] and have often involved interdisciplinary teams of computer scientists and clinical psychologists. De Choudhury et al [3] were proponents of supervised learning methods for predicting depression among populations. Exploiting post-level and user-level features from a crowdsourced Twitter dataset, they developed the social media depression index. To do this they used a support vector machine (SVM). The social media depression index could be used to determine the degree of depression manifested by users in their daily tweets. In a US demographic population study, they observed that women were 1.5 times more likely to express signs of depression on social media than men, which marginally exceeded findings from epidemiological surveys on formal diagnoses that suggest the figure to be 1.3 [109]. The overestimation was linked to the greater emotional expressivity of women [110], suggesting that methods more sensitive to language use could help develop more robust models. Such methods include topic modeling via latent Dirichlet allocation (LDA). While this approach has also been used for predicting depression in Twitter users [32], its results have to be taken cautiously as its dataset, in terms of users with depression and control users, was not deemed a representative sample of the population [38]. Later work used LDA-derived features as input to an SVM classifier to discern between users with depression and control users on Twitter [40]. Although the effectiveness of the topic-driven approach was demonstrated to some extent, only a modest result of 35% sensitivity was achieved. In a similar experimental setup for the prediction of depression in Twitter users [43], another traditional ML algorithm, random forests, was deployed using Linguistic Inquiry and Word Count (LIWC) features derived from post text. A commendable area under the receiver operating characteristic score of 87% was achieved and the method was validated by the collection of the mental health histories of its 204 participants via the Center for Epidemiologic Studies Depression Scale questionnaire. Tsugawa et al [40] acknowledged that emerging deep learning algorithms could well advance the methods in this area and were likely to inform future work. We explore these algorithms in the next subsection, Language Models and Transformers. A contemporaneous review also concluded that advances in NLP and ML were making the prospect of large-scale screening of social media for at-risk individuals a near-future possibility [42]. It also cited 2 studies that were influential in dataset design methods [31,32] that we discussed in the Datasets on Social Media and Mental Health section as being likely to help realize this.

By 2019, interest in methods for early prediction of depression had developed due to the recognition that they could help people receive the health care and social support they need sooner than they otherwise might [44]. Burdisso et al [44] designed an algorithm named SS3 that would calculate the degree to which some given text belonged to a certain category. While it could be generalized to any domain, in this case, it was used to classify depressed and control users of the longhand forum Reddit. It demonstrated superior early risk classification performance across several different experimental settings when compared to baselines computed using more traditional algorithms such as SVM. It also demonstrated significantly faster computation times; approximately 20 times faster than SVM. A further aim of SS3 was to provide explainability [111] for its classification decisions. It could display pertinent excerpts of a user’s Reddit text, such as “Fact is, I was feeling really depressed and wanting to kill myself,” which may assist clinicians. This transparency could not be gleaned from traditional “black box” algorithms such as SVM. SS3 was also hailed as a low-resource method, because, unlike SVM, it does not necessarily need to process the entire input text before returning its classification decision. However, it was acknowledged that because it examines each word of the input text in a singleton fashion, it would not consider potentially crucial 2-word phrases such as “kill myself” in a classification decision.

#### Language Models and Transformers

The capabilities of language models (LMs) had become well understood in NLP by the start of the 2020s. So, further to the work conducted by Burdisso et al [44], Bidirectional Encoder Representations from Transformers (BERT) and A Lite BERT (ALBERT) were deployed in an early depression prediction task involving tweets that denoted whether a user was with depression or anxiety or with no disorder [45]. As BERT and ALBERT necessarily consider the context of each word they encounter in a classification task, the consideration of n-word phrases is inevitable, thus addressing a matter highlighted by Burdisso et al [44]. In an experimental setting where users with depression and control users were balanced, an *F*_1_-score of 77% was achieved using BERT, compared to an SVM baseline of 65%. In an imbalanced dataset however, which is a more accurate representation of real-world scenarios where these tools could be applied, BERT achieved an *F*_1_-score of 74% compared to SVM’s score of 75%. Malviya et al [46] performed a similar experiment where individual posts in a Reddit dataset would be classified as depressed or nondepressed in nature by BERT and traditional baseline algorithms [46]. Once again, strong BERT performance was observed in a balanced experimental setting, therefore, strengthening evidence that further research is needed before LMs could be deployed for this prediction task in more realistic, imbalanced settings. Suggestions include generating synthetic instances to create balance [112] and resampling [113]. A review of deep learning approaches to mental health prediction [47] that postdates both studies [45,46] echoed the need for further work involving much larger datasets while acknowledging the impact of existing datasets that we have already highlighted [33,35].

Some of the most recent methods have harnessed generative AI, principally using GPT [104]. The arrival of generative AI has enhanced opportunities in this domain. We have already noted that the use of quality data is crucial in the pursuit of methods for predicting mental health status. Such data are often scarce and have given rise to data augmentation techniques [94,114]. A slightly different approach involves synthesizing data derived from existing data [115]. In an annual workshop task, a participating team used ChatGPT to synthesize data that would help develop models for identifying BDI-II-recognized depression symptoms conveyed in Reddit posts [48]. Several thousand apparently suitable texts were generated. For example, to the BDI-II response “I am so sad or unhappy that I can’t stand it,” ChatGPT formed the text “I’m so overwhelmed by sadness that I can barely function anymore.” However, it was found that models for linking such texts to appropriate BDI-II responses performed more strongly with respect to real data rather than their synthesized counterparts. It was suggested that the synthesized texts were overly detailed and complex, thus confounding LMs used in the subsequent classification exercise. One LM used was MentalRoBERTa [102], which is trained on real Reddit data. More judicious use of ChatGPT such that it produces less detailed texts that are more semantically similar to the BDI-II responses was proposed as follow-up work. A further use of a GPT has been in the automatic trisection [49] of the SetembroBR Twitter corpus of users with depression and control users [116]. The GPT was prompted to label each tweet as having either high, medium, or low relevance to mental health. The labeled dataset was then used as an input to a bag-of-words classifier and its prediction performance was compared with that of a BERT-derived baseline produced by an earlier study [117]. While this approach was markedly low resource and improved the baseline result by 5% in terms of sensitivity, it was acknowledged that improved prompting of the GPT, perhaps by using a more formal definition of depression, might see further improved sensitivity. Therefore, large LM (LLM) supported GPTs have shown potential for aiding mental health prediction in a variety of ways. For that potential to be fully realized, computer scientists need to consider how GPT prompting techniques can be optimized in each context.

#### Considerations for Schizophrenia

Finally in this section, we examine the literature’s coverage of schizophrenia. In a 2015 study by Mitchell et al [39], LDA was applied in a Twitter dataset with the goal of distinguishing between users with schizophrenia and controls. Key findings were that irrealis mood (denoted by the use of uncertain terms such as “think” or “believe”) [118] and flat affect (due to lack of emoticon use) [119] were prevalent in the posts of people with schizophrenia. A limitation of their dataset was that users’ self-statements of schizophrenia diagnoses could not be verified, which is a problem in this field of research as psychotic symptoms might preclude people from believing in their diagnoses [120,121]. In any case, people with schizophrenia may be reluctant to disclose their diagnoses on social media because they are likely to receive stigmatized responses [122,123]. Birnbaum et al [41] attempted more accurate identification on Twitter using a human-machine partnered approach. Self-reported schizophrenia statements were scrutinized for their authenticity by a psychiatrist and a graduate-level mental health clinician. The ML-derived model subsequently developed was able to distinguish between users with schizophrenia and controls with 87% sensitivity. Despite this, the authors acknowledged that truly confirming the diagnosis of a user who makes a self-disclosure statement is not possible without access to the user’s EHRs.

### Longitudinal Analyses of Mental Health

Studies discussed so far have tended to predict a person’s mental health at a particular point in time. However, a person’s mental health state is not static [34]. Indeed, it has been argued that inferences derived from sample-level “snapshots” of mental health states might not lead to reliable predictions of the individual-level variation in these states through time [124]. Therefore, research has also examined temporal profiles of mental health disorders and symptoms. A 2011 study considered US college students’ Facebook status updates and their potential for exhibiting content that may reveal symptoms of depression [24]. It was noted that opportunities for recognition and treatment of depression were being missed, particularly among college students [125,126]. Therefore, Facebook, a social media platform that had become well-established among the student population [127], presented innovative opportunities to identify college students at risk. A manual exercise saw the collection of Facebook status updates of 200 students that spanned 1 year. Human annotators then scrutinized each post, denoting a depressive symptom if deemed present according to the *Diagnostic and Statistical Manual of Mental Disorders* criteria [128]. A quarter of profiles exhibited at least 1 depressive symptom (as inferred through the use of terms like “hopeless” or “giving up”). This evidence that Facebook may allow the identification of at-risk students would be a precursor to future longitudinal analyses.

Schwartz et al [50] sought to gauge how the level of depression changes among Facebook users during a calendar year. Their method involved the extraction of 1-to-3-word terms, LDA-derived topics, and LIWC categories from the status updates of >28,000 users. A regression model was developed that indicated a significantly higher degree of depression among users during winter months than in summer months, which is compatible with observations made in the psychiatry literature [129]. A baseline model that considered only the average sentiment across each user’s status updates was outperformed in terms of accuracy almost 3-fold, although the optimal model only exceeded 30% [130]. By comparison, Loveys et al [51] conducted experiments predicting mental health statuses during much shorter time spans, hours in fact. Tweets belonging to >2500 users who self-stated a diagnosis of either anxiety or schizophrenia were automatically labeled with either positive, neutral, or negative sentiment. For each user, the changes (or otherwise) in terms of sentiment across 3 subsequent tweets that occur within any 3-hour window were observed. These observations were dubbed “micropatterns.” It was noted that users with schizophrenia were less likely to show emotional variability between tweets than control users, which perhaps demonstrates a deficit in affective expression, a known schizophrenia symptom [131]. Users with anxiety were less likely to make consecutive positive tweets than controls, again consistent with psychological findings [132]. However, the micropatterns did not contain sufficient details to indicate the severity of the mental health disorders but enriching the automatic labeling process by considering linguistic features other than sentiment (eg, terms that may be mapped to specific symptoms) may help in this respect.

Emotions and their changing nature over a series of web-based postings have also been studied. Seabrook et al [53] considered whether “emotion dynamics” in Twitter and Facebook may provide early indicators for depression risk. The feasibility of using emotion variability and instability as an indicator of depression severity, measured by the Patient Health Questionnaire-9 [133], was explored. It was hypothesized that self-reported depression severity would be positively associated with negative emotion word variability and instability across status updates. Status updates and depression severity ratings of 29 Facebook users and 49 Twitter users were collected. MoodPrism [134] would gauge the emotion of their status updates and the severity of depression (via Patient Health Questionnaire-9) over a 1-year period. Results suggested that instability in the negative emotion expressed on Facebook provides insight into the presence of depression symptoms for social media users. Also, greater variability of negative emotion expression on Twitter may, in fact, be protective for mental health. However, these observations were constrained by the users’ tweets being unavailable for manual inspection due to privacy reasons. Therefore, no manual verification was possible, and the results are essentially unreproducible. Another study from 2018 also considered emotion expressions on Twitter for their use in predicting depression [54]. In total, 8 basic emotions (anger, disgust, fear, happiness, sadness, surprise, shame, and confusion) were sought in the tweets of 585 users with depression across a 4-month period. The average intensity of each emotion was calculated via the EMOTIVE ontology [135] and used in a time-series analysis of each user. This analysis in turn helped build ML-based classifiers for labeling previously unseen Twitter users as being either depressed or not. In the best-performing setup with a random forests classifier, 87% sensitivity using temporal features was achieved compared to 71% using simple LIWC features. This suggests that the changes in an individual’s emotions over time show potential in identifying users with depression. Fine-grained consideration of the language used in tweets, such as tentative (eg, “maybe”) and temporal-related terms, may not only predict its presence but also its severity [56].

The emergence of transformer-based LMs coincided with the onset of the COVID-19 pandemic. It was no coincidence that interest grew in methods for monitoring population-level depression on social media at that time and that LMs would feature. In one study, tweets dated between March 3 and May 22, 2020, were collected regarding users who self-disclosed having depression [55]. The goal was to develop a model for monitoring the fluctuation of depression levels of different groups as COVID-19 propagated. Using the BERT-like model XLNet [136] and a geographical aggregation of users in the dataset, they demonstrated how depression levels fluctuated between the aforementioned dates in New York, California, Florida, and the United States as a whole. It was observed that depression levels in all 4 geographical areas were similar during the pandemic, with a steady increase after the announcement of the United States National Emergency on March 13, a modest decrease after April 23, followed by a steep increase after May 10. The overall depression score of Florida was substantially lower than the United States average and the other 2 states, possibly because it has a lower depression level overall compared to the average US level irrespective of the pandemic. These findings were constrained by the fact that only Twitter users were considered, who therefore are not fully representative of the population. In a further use of LMs, Owen et al [57] aimed to determine how far in advance of a Reddit user’s depression diagnosis their postings were most indicative of their condition. Overall, 56 users with depression and 168 controls were acquired from an intersection of the RSDD [33] and RSDD-Time datasets [34]. BERT and a specialist LM, MentalBERT [102], considered all user posts in increasingly large temporal bands up to 24 weeks (approximately 6 months) before the diagnosis dates of users with depression. The LMs achieved *F*_1_-scores of 0.726 and 0.715, respectively, when 12 weeks of postings were considered, suggesting therefore that the most poignant language used by users with depression occurs in the final 3 months before their eventual diagnosis. The reason for the specialist LM performing less effectively than its general counterpart may be explained by the fact that the former is trained on text found in mental health subreddits, and such postings are not included in RSDD. Findings were tempered by the fact that the diagnosis dates were mere estimates, as explained in the discussion of RSDD-Time in the Datasets on Social Media and Mental Health section. In any case, it was posited that a multimodal classification approach might provide more robust results. For example, a Reddit user’s upvotes or downvotes for posts may also be predictive of their mental health state.

We conclude this section by again exploring what the literature has covered in the realm of schizophrenia. Hswen et al [52] investigated the language used by Twitter users with schizophrenia to observe whether it would help assess suicide risk [52]. They examined the frequency of suicide-related tweets, paying particular attention to the times of such tweets. They hypothesized that Twitter users who self-identify as having schizophrenia would be significantly more likely to post tweets containing suicide terms when compared to Twitter users from the general population, thereby reflecting the elevated risk of suicide observed among individuals with schizophrenia in real-world settings. The tweets of 203 users with schizophrenia and 173 control users covering a 200-day period were collected. Only tweets that contained the words suicide or suicidal were targeted because, perhaps not surprisingly, the term suicide is frequently contained in suicide-related conversations [137,138]. Crucially, the time of day of each tweet was recorded. A logistic regression model predicted that the users with schizophrenia showed significantly greater odds of tweeting about suicide compared with control users (odds ratio 2.15, 95% CI 1.42-3.28). Considering the times of tweets, the frequency of conversations about suicide on Twitter correlated significantly with discussions about depression and anxiety, another trend that is consistent with established data [139,140]. However, similar to the studies discussed previously [39,41], the inability to be able to verify the diagnoses of the users with schizophrenia was cited as a main limitation.

### Ethical Aspects of the Data and Analysis of Mental Health

When constructing datasets, developing methods, and performing longitudinal analyses to aid mental health prediction, people’s privacy ought to be considered. In 2016, Mikal et al [59] sought to determine the attitudes of Twitter users toward the platform’s use in population health monitoring. Their qualitative study focused on depression. A focus group was formed of Twitter users, some of whom had previously received a diagnosis of depression while others had not. The group was canvassed for their opinions on the prospect of machine-driven health monitoring and their privacy expectations thereon. Broadly speaking, participants were supportive of the use of publicly available data for health monitoring activities, provided that the user identities were concealed. The concerns about the reliability of methods that use crude keyword searches and the misleading findings they could yield were also noted. An incorrect labeling of depression for a user whose identity is revealed would be considered stigmatizing according to participants. The study was only indicative because the group comprised just 26 Twitter users of a narrow demographic (predominantly male with an average age of 26.9 years). However, a concurrent study by Conway and O’Connor [60] gleaned further evidence of fears regarding such stigmatization.

Nicholas et al [62] address similar privacy matters. They note that the introduction of the General Data Protection Regulation in Europe and popular scandals such as Cambridge Analytica’s use of Facebook data brought data privacy into sharp focus. User concerns are many and varied. Some users fear that the research findings may affect credit card applications [141] and employment prospects and attract stigma [142]. Fears are compounded by evidence that deidentified data can be reidentified using materials published alongside research articles [143]. Indeed, the desire for anonymity appears particularly widely held, which echoes the findings by Mikal et al [59] and is reinforced by Vornholt and De Choudhury [63]. Therefore, obtaining explicit user consent for the use of their data is considered crucial. A possible route is via acceptance of social media platform terms and conditions. However, as these may not be read and understood [144], this may not constitute informed consent. One solution is to explicitly invite users to donate their social media data for research purposes [145]. Another proposal is a feature that enables users to opt in or out of their data being used as they post it [146].

A matter has also been identified regarding the terminology used in this area of mental health research. Chancellor et al [61] reviewed how human participants are referred to in literature for predicting mental health status using social media data. Common traits were seen across 55 articles. For example, introductions often refer to human participants as “individuals” and “people,” but technical sections then refer to them as “samples” and “data,” respectively. It is argued that this may present risks to scientific rigor and the populations the research aims to help. Inconsistent terminology may cause misunderstandings regarding study design thus affecting reproducibility of results. Depersonalization and dehumanization may be another byproduct [147]. This may cause individuals and communities to become stigmatized, echoing the findings of the studies discussed earlier. To alleviate this, it is suggested that more human-centered methods such as participatory design should be considered where interviews and field studies are conducted. However, this is at odds with the challenges highlighted in the Datasets on Social Media and Mental Health section where acquiring sizable datasets through such methods is largely intractable [32].

With respect to schizophrenia, Välimäki et al [58] determined via their review that the perceptions and risks of social media interventions are largely unexplored. However, there are suggestions that some clinicians fear that the use of web-based peer support without professional moderation may cause anxiety in the bearer of the disorder [148]. Cognitive deficits in people with schizophrenia can inhibit the development of digital skills [149], evidencing clinicians’ misgivings.

## Discussion

### Principal Findings

We have seen that there is growing interest in methods for predicting mental health status using social media data, particularly those that involve NLP. Enthusiasm has been notable since the COVID-19 pandemic when interest in remote monitoring of individual- and population-level mental states grew. Indeed, the search strategy followed for this review yielded more articles in the years 2020 to 2021 than in the previous 20 years; 917 and 903, respectively (Multimedia Appendix 1). Methods have progressed from those that use features from text as input to traditional ML algorithms, to increasingly sophisticated approaches using transformer-based LMs and now, LLMs. The research community has endeavored to provide social media data to support this work and to do so in ways that are increasingly sensitive to ethical and privacy concerns of the participants involved.

Our review has not only shown depression to be the most common condition reported in publicly available datasets, it also highlights the need for much larger samples where contextual information on this and other conditions, such as a date for the diagnosis and not just its presence, is denoted to a high degree of accuracy. Having such data would likely strengthen results found in longitudinal studies, most of which have focused on depression as well, providing more opportunities for predictions before an eventual diagnosis is formalized [57]. Obtaining such ground truth data via traditional confidential questionnaires is time-consuming and intrusive from the participant’s point of view [134]. A solution may involve obtaining consented access to EHRs to accompany the users’ social media postings, as piloted by Eichstaedt et al [150]. Indeed, this means of verification is crucial in studies that consider schizophrenia because diagnosis self-disclosure statements, although having high sensitivity [151], may lack specificity [120,121]. In any case, social media data obtained also needs to be broadened to better support NLP methods. For example, Reddit datasets should routinely include postings from mental health subreddits in addition to other subreddits [79]. This would help ensure that LMs pretrained on such data are less prone to biases that may dampen the effectiveness of methods developed thereon [80]. LLM-driven technologies, such as ChatGPT and its successors, will likely underpin methods in the immediate future. However, a fledgling attempt involving Reddit posts found that models were better able to detect BDI-II-measured depression symptoms using authentic data rather than LLM (GPT-3) synthesized data [48]. It was suggested that improved prompt manipulation is needed to produce synthesized data that are less stilted. Another role for LLMs may be in the automatic labeling of mental health dataset instances. Ramos dos Santos and Paraboni [49] produced evidence that an LLM (GPT-3.5) can perform promisingly (72% sensitivity) when distinguishing between tweets of users who may have depression and users who likely do not. LLMs may eventually offer a far less costly alternative to dataset labeling than manual approaches. Psychiatry literature suggests LLM performance in these settings could be improved by prescribing potentially time-consuming trials to learn what prompts are best suited for specific tasks [152,153]. Instruction fine-tuning is one such proposal for improving LLM performance. LLMs including GPTs are trained on very large, nondomain specific datasets such as Wikipedia. However, further training an LLM on smaller, domain-specific datasets may enhance its performance in that domain. For example, when comparing the performance of a nonfine-tuned LLM and its fine-tuned counterpart, Xu et al [154] measured a 23.4% increase in accuracy across 6 different mental health prediction tasks involving Reddit data. However, fine-tuning ought to be performed using a wider range of domain-specific datasets, which is advisable to reduce biases in the resulting LLM.

With respect to population-level and individual-level longitudinal studies, we found the analysis of emotions conveyed in social media posts to be an underrepresented topic of research in this area. Consideration of fine-grained language features may also help to better predict depression severity over time [56]. In fact, the most promising approaches will probably involve those that augment NLP; multimodal methods that consider nontext features from social media activities are expected to help provide richer findings. In Twitter, this may involve consideration of user geolocations and profile images. For example, Ghosh et al [155] attempted to distinguish between users with depression and users without depression by considering their profile images and the text of their profile descriptions. A classifier that used features from the profile image outperformed a baseline classifier that used only features from the profile description by approximately 10% in terms of the *F*_1_-score. While profile images may be predictive of users’ mental health statuses to an extent, there are confounding factors that these multimodal methods must address. For example, people with depression are likely on social media platforms to display positive-looking pictures (including profile images) as opposed to negative-looking ones, according to Ghosh et al [155]. This perhaps counterintuitive phenomenon has been dubbed “smiling depression” and training of multimodal models with larger, labeled datasets is needed so that they may become more discerning in these conditions. Semwal et al [156] have also evidenced in similar experimental settings that information contained in tweet text and profile images complement one another and ought to be used in alliance. They recorded that their multimodal model outperformed their best-performing textual and image-only models by 3.5% and 27.1%, respectively, in terms of *F*_1_-score. Therefore, the conclusion was that images seem to contain significant information regarding a user’s mental health status, thus motivating further study in mental health status prediction. Meanwhile in Reddit, multimodal methods may involve time-aware consideration of user posts. One study considered the relative time between posts as a feature for distinguishing between Reddit and Twitter users with and without depression [157]. Obtaining an *F*_1_-score of 0.93 with Reddit and 0.87 with Twitter, it was concluded that a time-aware approach to classification is more effective where posting frequency is relatively high. The supposition is that the concise nature of Twitter posts, compared to the often much lengthier posts on Reddit, contributes to users posting more frequently on Twitter. A further study considered a multimodal approach with emphasis on emojis, again in the task of distinguishing between users with and without depression on both Twitter and Reddit [158]. With *F*_1_-scores of 0.80 and 0.95 being achieved for Twitter and Reddit, respectively, it could be concluded, given the 2 studies that have just been outlined, that different multimodal approaches will be suitable for different platforms.

The advent of multimodal approaches may also help allay a privacy-related concern that our review has brought to the fore. The public has expressed concerns about methods for predicting mental health status that harness primitive keyword searches due to the risk of unreliable output. Naturally, a social media user may be affronted at receiving an incorrect diagnosis of depression, anxiety, or schizophrenia [59,60]. Multimodal approaches that more accurately capture people’s real-life behaviors are thus being pursued [159]. It is not only methods that need to improve to gain public confidence; more fundamentally, the means of collecting data for use in any study need to be more explicit and have user consent. Inviting users to grant access to their social media data for research purposes on a large scale, perhaps at the point that they publish a social media posting, could become widespread [160]. However, such invitations must be accessible to a wide demographic. Privacy literacy, which describes one’s understanding of the risks of sharing information on social media, is considered more prevalent among women than men, for example [161].

Finally, our literature search returned many articles that consider the effects of social media use on a user’s levels of depression and anxiety (Multimedia Appendix 1). A primary hypothesis, greatly debated in specialist literature [162], is that extended or otherwise distinct patterns of social media use may cause or exacerbate these mental health disorders. This was not the subject area of this study, but our results on the volume of published articles suggest that this related matter perhaps merits a review of its own.

### Potential Clinical Applications

With reference to the research covered in this review, we now consider the potential clinical applications of using AI on social media data. These include (1) evaluating data at a population level to inform health care delivery and policy making, (2) identifying and providing access to support and interventions for those at risk of developing mental health problems, and (3) monitoring existing individual patients to detect and intervene at early signs of relapse [163]. The third application area was underrepresented in methods for predicting mental health status literature.

At a population level, AI and NLP may be used to navigate large volumes of data to inform clinical needs in a particular area, to identify changing patterns of mental illness across populations and time, to better understand patients’ experiences and perceptions of health services, and to identify patterns of risky behaviors among certain demographics (eg, young people accessing accounts linked to proanorexia or encouraging self-harm). As noted earlier, NLP was used to evaluate large volumes of social media data during the COVID-19 pandemic and identify the specific concerns of people living with mental illness, including health anxieties, loneliness, and suicidality [164]. This type of information can be used to inform resource allocation in health services and the development of government policies. Crucially, this analysis can be performed relatively quickly (particularly compared to traditional research methods), which is essential during periods of instability, such as a public health crisis, where decisions need to be made rapidly.

At an individual level, AI may be used to identify people at risk of or living with mental health problems and enable organizations to provide early intervention support. There are some concerns regarding consent, data use, and privacy, as noted in the Ethical Aspects of the Data and Analysis of Mental Health section. Interestingly, while both young people and mental health professionals somewhat agree that social media companies should use AI to proactively detect users at risk of suicide or self-harm and signpost them helpful information and resources, they felt more strongly that AI capabilities should be used to promote helpful content such as psychoeducation [165]. In addition, there are logistical challenges to doing this, such as how individual data collected by global platforms can be harnessed by localized health care providers to support care.

Despite these challenges, social media has been proven to be a useful tool to identify relevant individuals for research, including delivering interventions to young people living with eating disorders [166] and who have been exposed to suicide (eg, a friend or family member had died by suicide or attempted suicide) [165] and using Facebook data to detect relapse in patients with schizophrenia [167]. As an example, Birnbaum et al [167] used LIWC on extracted Facebook archives and concurrent medical records for participants with psychosis. Researchers built an individual-centric classifier to predict re-admission to the hospital due to exacerbation of psychotic symptoms. However, the sensitivity of the prediction model was low (38%) indicating that the algorithm only identified a small proportion of all those who relapsed. Furthermore, the algorithm was applied to retrospective Facebook archives and paired with retrospective medical records, all with explicit consent from participants. The use of social media data to prospectively predict relapse in patients is likely to be considerably more challenging. As the authors noted, patients may change their social media behavior if they are aware that they are being actively monitored by their care team.

While the AI-driven mental health status prediction methods outlined may appear to lend themselves readily for use in clinical practice, there are limitations that need to be addressed before they are adopted. A chief limitation, as already mentioned in this review, is the likelihood of bias in methods based on data that do not represent diverse populations [168,169]. Thus, they may not be able to account, for example, for the fact that mental health conditions may present differently in different people. This is challenging to overcome because the field of mental health care is limited in its access to large, high-quality datasets. Compounding these limitations is the fact that the underlying biological processes of mental health disorders are still poorly understood meaning that models must be bootstrapped from observations rather than be derived from first principles. Indeed, the nature of decision-making in mental health care can be far more complex than that of other clinical areas. Indicative of this is the fact that the specific and objective task of tumor identification from an image is already successfully supported by AI-driven methods [170]. Mental health care therefore desires AI-driven methods that are transparent, explainable, and able to provide guidance to clinicians [26,169,171].

### Limitations

We have reviewed the literature in what we deemed 4 chief areas in the realm of predicting mental health status. There are opportunities for greater depth of coverage in these areas and they could be the subject of review articles of their own. There is also scope for a greater breadth of coverage that could fuel follow-up studies. For example, our coverage has primarily considered research related to NLP, with occasional deference to multimodal alternatives. Visual computing provides techniques applicable to data from predominantly image-based platforms, such as Instagram [6,9]. Experts in computer vision may therefore be able to provide greater insight here.

Being a narrative review, the nature of article selection and analysis is somewhat subjective. To mollify this, we used a well-defined search and selection process that borrowed features often used in systematic reviews [29] (Methods section).

In addition, we only considered articles in which the participants of the studies self-reported a diagnosis of depression, anxiety, or schizophrenia; however, more widely any sort of information garnered from a social media posting should be treated like a self-report. While this confers a certainty that the input reflects the experiences and beliefs of the social media user, providing the opportunity to automatically accrue large datasets that have information about mental health statuses, this approach also has weaknesses that have been explored in the psychopathological literature [172]. For example, compared to a manually compiled and curated dataset, there are likely to be more false positive instances of nearly any common diagnosis, although there may also be false negative instances or controls that do in fact bear a mental health disorder [32] are also possible. In the case of schizophrenia, the condition itself might be partly responsible for the unreliability of self-reports, creating an even larger weakness for automatically constructed datasets as previously highlighted.

We should also mention that the social media platforms covered in this review, including Facebook, Twitter, and Reddit, are ostensibly English-language platforms. This coverage is perhaps by virtue of our literature search and selection strategy, which excluded non-English language articles. Therefore, we acknowledge that the findings presented in this paper may well not apply to non-English language platforms such as Weibo [173] and VK [174], which are Chinese and Russian language platforms, respectively. A complementary narrative review that considers social media platforms concerning these languages and cultures could form future work.

Finally, we highlight a theme that has recurred throughout this review, which is that of biases in predicting mental health status research. Addressing these biases, or at least being aware of them, is crucial for ensuring accurate and generalizable findings. This review has concerned predominantly English-language social media platforms, which in turn, largely reflect Western culture. Therefore, when such findings are reported in the literature it must be ceded that they might not generalize to social media platforms that predominantly reflect Eastern culture. In any case, there are other platform-related biases that must be considered; certain platforms may be used largely by certain demographics. We have already noted that on platforms such as Facebook, Twitter, and Instagram, male users are in the majority [81] and that social media users are generally well-educated and affluent [82]. Cohort-based strategies for dataset construction have been trialed to account for these biases [36]. There are also user-oriented biases that may distort datasets. A user’s posting habits may change over time and convey a distorted view of their life and experiences [50]. This behavior may be influenced by reports published in traditional print media on the negative consequences of social media use [175]. It may also be influenced by the proliferation of use-limiting tools, which encourage users to choose carefully the personal information they share on social media platforms [176]. On a collective scale, certain users may post content significantly more frequently than others, creating imbalances in datasets and subsequent models. This is evident in 2 of the datasets we have covered [86,90]. Data augmentation is one approach that may alleviate this problem [94,114], while another includes data synthesis via LLMs [48]. Lastly, we should mention confirmation bias, which involves people’s tendency to seek data that support their beliefs and ignore or distort data contradicting them [177]. Wherever possible, a selection of appropriate datasets ought to be used in experimental setups so that conclusions are better balanced. In general, it is suggested that future research in the domain of mental health status prediction should seek and report data biases to enhance the reliability of findings [27].

### Conclusions

The research area of predicting mental health status is receiving much attention, particularly in recent years. The COVID-19 era appears to have been the catalyst for the expanding interest. Further work needs to be completed with respect to methods for predicting mental health status before they may be considered sufficiently reliable for clinical purposes. We have documented public misgivings about text-only approaches, particularly those that rely on keyword searches. We have also acknowledged that image-based social media platforms such as Instagram are in wide use. Therefore, to help gain public confidence, methods will likely need to be multimodal. That is, they will need to generalize to text-, voice-, image-, and video-based social media data. The pursuit is merited to help relieve strain on health care and mental health services. In fact, the integration of automated early health care intervention methods and traditional methods may be advantageous.

This work cannot take place in a vacuum; however, due consideration must be given to the ethical concerns regarding the collection and use of social media users’ data. Consent from users needs to be sought, perhaps by providing them with the opportunity to donate their social media data or by allowing them to choose to share their data for research purposes on a post-by-post basis. In any event, the purposes of collecting such data ought to be made clear to users through transparent data use agreements. Then, when data are subsequently compiled into datasets for public release, anonymization of the user accounts they contain is essential.

## Appendix

Multimedia Appendix 1 Verbose exposition of the 4-stage literature selection process.

## Abbreviations

AI: artificial intelligence
ALBERT: A Lite Bidirectional Encoder Representations from Transformers
BDI-II: Beck Depression Inventory-II
BERT: Bidirectional Encoder Representations from Transformers
EHR: electronic health record
LDA: latent Dirichlet allocation
LIWC: Linguistic Inquiry and Word Count
LLM: large language model
LM: language model
ML: machine learning
NLP: natural language processing
RSDD: Reddit Self-reported Depression Diagnosis
SMHD: self-reported mental health diagnoses
SVM: support vector machine

## Data Availability

Data sharing is not applicable to this paper as no datasets were generated or analyzed during this study.
